# Impact of Chromatin Dynamics and DNA Repair on Genomic Stability and Treatment Resistance in Pediatric High-Grade Gliomas

**DOI:** 10.3390/cancers13225678

**Published:** 2021-11-12

**Authors:** Lia Pinto, Hanane Baidarjad, Natacha Entz-Werlé, Eric Van Dyck

**Affiliations:** 1 DNA Repair and Chemoresistance, Department of Oncology, Luxembourg Institute of Health, L-1526 Luxembourg, Luxembourg; Lia.pinto@lih.lu (L.P.); Hanane.baidarjad@lih.lu (H.B.); 2 Faculty of Science, Technology and Medicine, University of Luxembourg, L-4365 Luxembourg, Luxembourg; 3 UMR CNRS 7021, Laboratory Bioimaging and Pathologies, Tumoral Signaling and Therapeutic Targets, Faculty of Pharmacy, 67401 Illkirch, France; Natacha.Entz-Werle@chru-strasbourg.fr; 4 Pediatric Onco-Hematology Unit, University Hospital of Strasbourg, 67098 Strasbourg, France

**Keywords:** pediatric high-grade gliomas, chemoresistance, chromatin dynamics, DNA repair, genomic instability, variant H3.3 histone, *ATRX*, DAXX, (peri)centromere, telomere, telomerase, alternative lengthening of telomere (ALT), synthetic lethality

## Abstract

**Simple Summary:**

Pediatric high-grade gliomas (pHGGs) are the leading cause of mortality in pediatric neuro-oncology, due in great part to treatment resistance driven by complex DNA repair mechanisms. pHGGs have recently been divided into molecular subtypes based on mutations affecting the N-terminal tail of the histone variant H3.3 and the *ATRX*/DAXX histone chaperone that deposits H3.3 at repetitive heterochromatin loci that are of paramount importance to the stability of our genome. This review addresses the functions of H3.3 and *ATRX*/DAXX in chromatin dynamics and DNA repair, as well as the impact of mutations affecting H3.3/*ATRX*/DAXX on treatment resistance and how the vulnerabilities they expose could foster novel therapeutic strategies.

**Abstract:**

Despite their low incidence, pediatric high-grade gliomas (pHGGs), including diffuse intrinsic pontine gliomas (DIPGs), are the leading cause of mortality in pediatric neuro-oncology. Recurrent, mutually exclusive mutations affecting K27 (K27M) and G34 (G34R/V) in the N-terminal tail of histones H3.3 and H3.1 act as key biological drivers of pHGGs. Notably, mutations in H3.3 are frequently associated with mutations affecting *ATRX* and DAXX, which encode a chaperone complex that deposits H3.3 into heterochromatic regions, including telomeres. The K27M and G34R/V mutations lead to distinct epigenetic reprogramming, telomere maintenance mechanisms, and oncogenesis scenarios, resulting in distinct subgroups of patients characterized by differences in tumor localization, clinical outcome, as well as concurrent epigenetic and genetic alterations. Contrasting with our understanding of the molecular biology of pHGGs, there has been little improvement in the treatment of pHGGs, with the current mainstays of therapy—genotoxic chemotherapy and ionizing radiation (IR)—facing the development of tumor resistance driven by complex DNA repair pathways. Chromatin and nucleosome dynamics constitute important modulators of the DNA damage response (DDR). Here, we summarize the major DNA repair pathways that contribute to resistance to current DNA damaging agent-based therapeutic strategies and describe the telomere maintenance mechanisms encountered in pHGGs. We then review the functions of H3.3 and its chaperones in chromatin dynamics and DNA repair, as well as examining the impact of their mutation/alteration on these processes. Finally, we discuss potential strategies targeting DNA repair and epigenetic mechanisms as well as telomere maintenance mechanisms, to improve the treatment of pHGGs.

## 1. Introduction

Despite their low incidence, pediatric high-grade gliomas (pHGGs), including diffuse intrinsic pontine gliomas (DIPGs), are the leading cause of mortality in pediatric neuro-oncology. Advances in the molecular profiling of pHGGs have led to their recent classification in distinct subgroups characterized by differences in tumor localization, clinical outcome, as well as concurrent epigenetic (i.e., DNA methylation pattern) and genetic alterations [1,2,3]. Central to this novel classification is the discovery of recurrent, mutually exclusive mutations affecting K27 (K27M) and G34 (G34R/V) in the N-terminal tail of histones H3.3 and H3.1, which act as key biological drivers in a large subset of pHGGs [4,5,6,7]. The K27M and G34R/V mutations are found predominantly in midline and hemispheric tumors, respectively. The K27M mutation impacts H3K27 methylation/acetylation as well as the methyltransferase activity of the enhancer of teste homologue 2 (EZH2) subunit of the polycomb repressive complex 2 (PRC2) acting at H3K27. On their part, the G34R/V mutations affect the activity of the SETD2 histone methyltransferase acting at H3K36. This leads to distinct epigenetic reprogramming, telomere maintenance mechanisms, and oncogenesis scenarios [1,8,9].

In contrast to H3.1—a canonical replicative histone expressed during S phase and deposited on newly replicated DNA by the histone chaperone CAF1—the histone variant H3.3 is expressed throughout the cell cycle and deposited by different histone chaperones independently of DNA replication. H3.3 is enriched at genomic sites undergoing active nucleosome turnover (i.e., gene bodies and DNA regulatory elements associated with transcriptional activity) where it is deposited by the histone regulator A (HIRA) histone chaperone. H3.3 is also found in heterochromatin compartments whose structural integrity is paramount to prevent genomic instability, such as (peri)centromeres, telomeres, and transposons, where H3.3 is deposited by a histone chaperone complex composed of alpha-thalassemia/mental retardation syndrome X-linked (*ATRX*) and death domain-associated protein (DAXX) [10,11,12,13,14]. Notably, nearly all pHGG tumors bearing H3G34R/V mutations and a substantial number of H3K27M-mutant tumors also contain mutations in *ATRX*/DAXX [4,15]. At the protein levels, *ATRX* is undetectable in biopsies from patients harboring *ATRX* mutations [4]. In addition, mutations in H3.3 and *ATRX*/DAXX are frequently associated with an alternative mechanism of telomere maintenance relying on DNA recombination called ALT (alternative lengthening of telomeres) [4]. 

For pHGG patients with sus-tentorial locations, radiotherapy follows surgical resection, which tends to be extensive, and is associated at least with DNA alkylating agents like temozolomide (TMZ) [16]. In thalamic pHGGs and DIPGs, targeted therapies are nowadays proposed concomitantly to irradiation and prolonged until progression [17,18,19,20,21]. However, contrasting with our understanding of their molecular biology, there has been little improvement in the treatment of pHGGs where the development of tumor resistance driven by complex DNA repair pathways severely limits the efficiency of genotoxic chemotherapy and ionizing radiation (IR) [21,22].

In this review, we summarize the major DNA repair pathways that contribute to the resistance to current DNA damaging agent-based therapeutic strategies and describe the telomere maintenance mechanisms encountered in pHGGs. We then review the functions of H3.3 and its chaperones in chromatin dynamics and DNA repair, as well as examining the impact of their mutation/alteration on these processes. Finally, we discuss potential strategies targeting DNA repair and epigenetic mechanisms as well as telomere maintenance mechanisms, to improve the treatment of pHGGs.

## 2. DNA Repair Mechanisms Promoting Resistance to IR and Alkylating Agent Therapy

Complex, often redundant DNA repair mechanisms orchestrated by the DNA damage response (DDR) [23] operate to repair DNA lesions introduced by radiotherapy and genotoxic chemotherapy or stemming from endogenous sources such as the collapse of replication forks associated with replication stress [24]. These include direct repair mechanisms, as well as the base excision repair (BER) pathway, the nucleotide excision (NER) pathway, the mismatch repair (MMR) pathway, and pathways that repair DNA single-strand breaks (SSBs) and double-strand breaks (DSBs). The genes encoding the components of the various DNA repair machineries form a long list (ref [25], see https://www.mdanderson.org/documents/Labs/Wood-Laboratory/human-dna-repair-genes.html#Human%20DNA%20Repair%20Genes (accessed on 18 October 2021) for an updated table maintained by Wood and Lowery). To this list, one must add the hundreds of chromatin factors that modulate DDR and DNA repair within the chromatin landscape [26]. 

**Figure 1:**
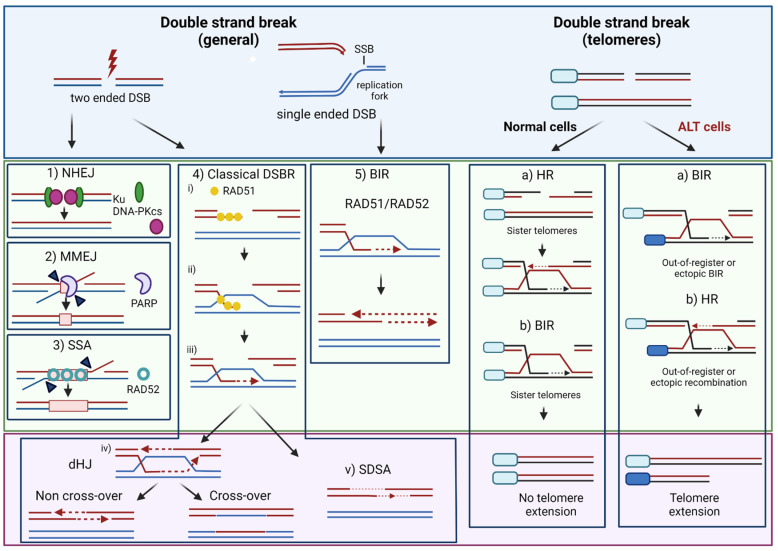
Schematics of the major DSB repair pathways. In NHEJ, the broken ends are bound by the Ku heterodimer, leading to the recruitment and activation of DNA-dependent protein kinase catalytic subunit (DNA-PKcs) and subsequent juxtaposition of the ends, followed by an eventual processing step—for instance, in the case of “dirty” ends produced by IR [33]—to generate ligatable ends. Central to HR, SSA and MMEJ is a DNA end resection step [34] that generates 3′-ended, single-stranded DNA (ssDNA) tails. In MMEJ, a pathway that is promoted by poly(ADP-ribose) Polymerase 1 (PARP1) acting in part to prevent Ku binding to DSBs [35], end-resection exposes short regions of complementary sequences (microhomologies) that facilitate end-bridging before processing the non-homologous flaps by the XPF/ERCC1 endonuclease (arrows), filling-in DNA synthesis and ligation. In SSA, homologous sequences exposed by long ssDNA strands are annealed by the RAD52 recombinase [36] to generate an intermediate with non-homologous flaps that are clipped by XPF/ERCC1, allowing filling-in DNA synthesis and ligation [37]. In DSBR, The RAD51 recombinase acts on long resected tails to assemble nucleoprotein filaments (i) that invade the homologous donor sequence to generate a displacement loop (D-loop) (ii) and prime repair DNA synthesis at the invading end, templated by the homologous sequence (iii) [31]. DSB repair can then proceed via classical DSBR (also called gene conversion) or synthesis-dependent strand annealing (SDSA). In DSBR, capture of the second end by the displaced strand of the D-loop is associated with extended DNA repair synthesis and the formation of a double Holliday junction (dHJ) (iv) whose resolution/dissolution can lead to non-crossovers or crossovers between the sister chromatids. On the other hand, SDSA involves limited DNA synthesis as well as the displacement of the invading strand from the donor sequence and its annealing to the second, unengaged resected end, thereby preventing dHJ formation and crossover products. Single-ended DSBs are formed when the replication fork collapses upon an encounter with, e.g., SSBs or trapped protein-DNA complexes. The repair of seDSBs can occur through HR or BIR mechanisms mediated by the RAD51 or RAD52 recombinase. BIR is initiated by strand invasion to form a D-loop and progresses via D-loop migration. Of note, recent studies suggest that RAD51-dependent BIR can also take place to repair two-ended DSBs in mammals, as previously shown in yeast [38,39]. BIR mechanisms and HR (DSBR and SDSA) can also take place at telomeres. In cells with intact *ATRX*/DAXX, the model of Lovejoy et al. [40] proposes that telomere cohesion promoted by *ATRX* fosters “in-register” HR/BIR using the sister telomere, while loss of *ATRX* promotes “out-of-register” or ectopic HR/BIR, resulting in a net increase in telomere length, as observed in ALT cells. The authors further propose that disruption of a DAXX-dependent function of *ATRX* promotes BIR over other pathways [40]. See text for details. For the sake of clarity, only a few of the DNA repair factors involved in DSB repair have been indicated. Created with BioRender.

DSBs represent the most lethal form of DNA lesions induced by ionizing radiation and chemotherapeutic genotoxicants [27]. DSB repair can occur through homologous recombination (HR), itself subdivided in classical DSB repair (DSBR, also called gene conversion), synthesis-dependent strand annealing (SDSA) and break-induced replication (BIR) pathways, or error-prone pathways including single-strand annealing (SSA), non-homologous end-joining (NHEJ) and microhomology-mediated end joining (MMEJ, also called alternative end-joining) [28,29,30,31,32] (Figure 1).

**Figure 2:**
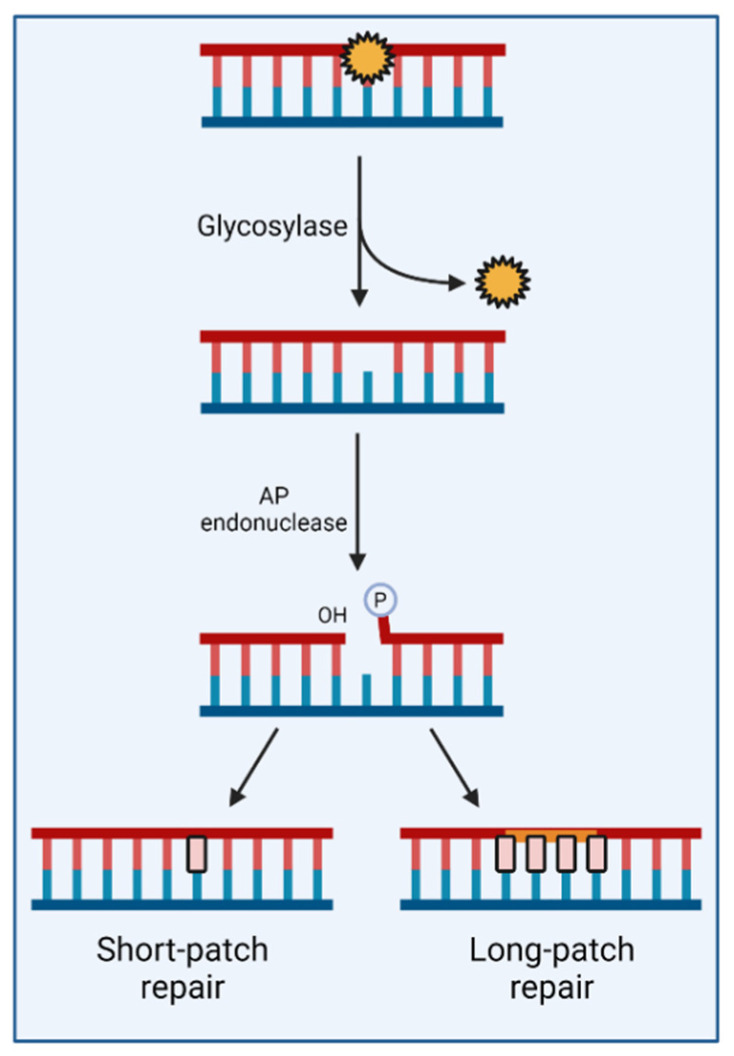
Salient features of the base excision pathway. BER is initiated by a DNA glycosylase (e.g., OGG1, NTH1, NEIL1-3, UDG, MPG) that recognizes specific types of base damage and mediates the excision of the damaged base, creating an apurinic/apyrimidinic (AP) site. Cleavage of the phosphodiester backbone by AP endonuclease 1 (APE1) then generates an SSB intermediate with a 3′-OH and 5′-deoxyribose phosphate (5′dRP) residue which is processed to allow nucleotide replacement by repair DNA synthesis in steps that can involve the replacement of either a single nucleotide (short patch BER) or several nucleotides (long patch BER). Of note, certain DNA glycosylases are bifunctional, possessing an AP-lyase activity that can process the AP site. BER mediates the repair of the major lesions induced by TMZ (N7-methylguanine and N3-methyladenine). It also provides the major mechanism for the removal of oxidative damage lesions. The repair of SSBs (not illustrated here), which involves their recognition by PARP1, is considered a subpathway of BER. Created with BioRender.

The majority of DNA-methylation lesions elicited by TMZ are repaired through base excision repair (BER)—a pathway also in charge of handling oxidative DNA damages such as 7,8-dihydro-8-oxoguanine (8-oxoG), thymine glycol, and abasic sites (Figure 2) [41]—or direct removal mechanisms catalyzed by the DNA demethylases ALKBH2/3 [42]. The most cytotoxic lesion induced by TMZ, O^6^-methylguanine (O^6^-meG), is removed by the DNA repair protein O^6^-methylguanine-DNA methyltransferase (*MGMT*) [43]. *MGMT* defines a direct repair mechanism as it reverses O^6^-meG lesions in a suicidal reaction whereby the methyl group of the damaged base is transferred to a cysteine residue in the active site of *MGMT* [42,43,44]. In adult glioblastoma (aGBM), silencing of *MGMT* through gene promoter methylation constitutes a crucial clinical biomarker [45]. In pHGG, both the frequency and importance of *MGMT* silencing remain controversial [46], although *MGMT* promoter methylation is observed at a high frequency (75%) in hemispheric pHGGs [47]. Unrepaired O^6^-meG lesions generate O^6^-meG/thymidine mismatches during S phase. These mismatches are recognized, but not resolved, by the MMR, resulting in persistent ssDNA gaps that cause replication fork collapse and DSBs [48,49,50]. 

The DSBs generated upon replication fork collapse are of a special type called single-ended DSB (seDSB) in opposition to the classical, two-ended DSBs generated, e.g., by IR (Figure 1). Notably, seDSBs can also occur when replication forks collide with base damage or SSBs generated as intermediates of the BER pathway [51], such as DNA:RNA hybrids [52], or protein-DNA complexes such as those trapped by the topoisomerase I poison camptothecin (CPT) [53], or the poly(ADP-ribose) polymerase 1 (PARP1) inhibitor olaparib [54]. The recent years have thus witnessed great interest in the molecular mechanisms underlying seDSB repair [55,56]. As for two-ended DSBs, seDSBs can be processed by HR or end-joining mechanisms. RAD51-mediated HR plays a central role in replication fork repair during the S and G2 phases of the cell cycle [57] through a recombination-dependent DNA replication pathway called break-induced replication (BIR) [58] (Figure 1). In cancer cells undergoing replication stress, a RAD52-dependent BIR pathway has been described [59]. RAD52-mediated BIR also promotes mitosis DNA synthesis (MiDAS) at common fragile sites, a process where RAD51 is dispensable [60]. NHEJ-mediated processing of seDSBs is toxic as it involves the juxtaposition and ligation of distant DNA ends, resulting in chromosomal aberrations and genetic instability [30]. However, fully active HR outcompetes NHEJ for the repair of seDSBs in S/G2 [61]. Several studies have underlined the involvement of HR in the repair of lesions resulting from O^6^-meG adducts [62,63,64]. 

Finally, a noted actor in DNA repair and target for DNA repair-based therapeutic approaches is PARP1, whose roles encompass DNA damage recognition and the recruitment of scaffolding proteins such as X-ray repair cross-complementing protein 1 (XRCC1) or other key DNA repair factors. PARP1 is central to SSB repair, BER, NER and MMEJ. It also contributes to DSB repair, stabilization of DNA replication forks, and the modulation of chromatin changes during DNA repair [65,66]. Importantly, the clinical use of PARP1 inhibitors such as olaparib in the treatment of HR-defective ovarian or breast tumors represents a paradigm for synthetic lethality [67]. The proposed mechanism of action of PARP1 inhibitors relies on the trapping of PARP1 on SSBs, leading to replication fork collapse and seDSBs that are funneled toward NHEJ in HR-defective cells, leading to genetic instability and cell death [65]. 

## 3. Telomere Maintenance Mechanisms

Telomeres, the physical ends of our chromosomes, are constituted of non-coding duplex TTAGGG repeats (between 9 and 15 kb in size) that terminate with a single-stranded, G-rich overhang (about 50 to 300 n in size) [68]. Telomeres can fold back on themselves, leading to the invasion of the duplex telomeric DNA by the single-stranded overhang and the formation of a telomere loop (t-loop) [69]. In addition, a complex of six proteins (TRF1, TRF2, POT1, TIN2, TPP1, and RAP1) called shelterin cap telomere ends, generating a nucleoprotein complex that protects chromosome ends from degradation and from being detected as DSBs [68,70]. Also involved in telomere maintenance and protection is a class of long, non-coding RNAs (lncRNAs) called TERRA (telomeric repeat-containing RNA) which are transcribed by RNA polymerase II from subtelomeres towards the telomeric repeat tracks, using the telomeric C-rich strand as a template [71]. Although they are not restricted to telomeres [72], a substantial proportion of TERRA transcripts remains associated with telomeres where they are an integral part of the telomeric heterochromatin structure [73,74]. Documented roles for TERRAs at telomeres include heterochromatin formation through the recruitment of factors such as heterochromatin protein 1 (HP1), histone methyltransferases and shelterin components [75], telomere protection [72,75,76,77], telomere replication [78,79] and downregulation of telomerase [72]. Of note, Montero et al. have shown that CRIPSR/cas9 deletion of 20q-TERRA—a major TERRA locus in human cells—elicited a loss of cellular viability in a battery of cancer cell lines [76]. 

Responsible for telomere replication is a nucleoprotein reverse transcriptase called telomerase that extends telomeres using an RNA template [80]. Active in normal stem cells, telomerase undergoes strong negative regulation in all other cell types [80,81]. However, activation of a telomere maintenance mechanism (TMM) is crucial to enable limitless replicative potential during tumorigenesis [82]. Two distinct TMMs can be observed in cancer cells: the reactivation of telomerase and the alternative lengthening of the telomeres (ALT) pathway. Whereas reactivation of the telomerase [83] is the major TMM taking place in adult glioblastomas [84,85,86,87], pHGGs are more prone to develop ALT [88].

### 3.1. Telomerase Reactivation

Telomerase activation is primarily achieved through hotspot mutations in the promoter of the *TERT* gene (*TERTp*) encoding the catalytic subunit of the telomerase [89,90]. *TERTp* mutations are rare in pHGGs ([91], and references therein). In addition, hypermethylation of a CpG-rich region within the *TERT* promoter—the THOR region (*TERT* hypermethylated oncological region)—which participates in *TERT* activation [92], is observed in pHGGs [93]. 

### 3.2. Alternative Lengthening of Telomeres (ALT)

In the telomerase-independent ALT pathway, which is frequently observed in pHGGs bearing driver mutations on H3.1/H3.3 and showing concomitant loss-of-function of the DAXX/*ATRX* complex [4], telomeres are maintained through DNA synthesis driven by homology-directed repair [94,95,96,97] (Figure 1). Although ALT telomeres retain duplex TTAGGG repeats with single-stranded, G-rich overhangs, as well as shelterin and other telomere-associated proteins, TERRA lncRNAs and the ability to form t-loops, they also exhibit specific features. These include (i) highly heterogeneous telomere length; (ii) presence of extrachromosomal telomere repeats including double-stranded telomeric circles (t-circles), partially single-stranded circles (C-circles and G-circles), linear duplex and more complex structures; (iii) the localization of telomeric DNA and associated proteins to nuclear promyelocytic leukemia (PML) bodies, referred to as ALT-associated PML bodies (APBs) and; (iv) telomere sister chromatid exchanges (tSCEs) [94].

Knowledge of the molecular mechanisms and repair factors responsible for telomere DNA synthesis and maintenance in ALT cells has emerged [94,95,96,97,98]. The evidence indicates that ALT is triggered by replication stress-associated telomere breaks and proceeds within APBs via homology-directed repair mechanisms including BIR [98,99,100,101,102]. In addition to RAD51-dependent HR pathways [103,104], at least two RAD51-independent BIR pathways have been reported to drive ALT, which differ in their requirement for the RAD52 recombinase [100,101,102,105]. Notably, recent evidence from Silva et al. indicates that TERRA transcription destabilizes telomere integrity in ALT cells, as assessed by the accumulation of γH2A foci (a DSB marker), thereby providing a trigger for ALT-mediated telomere elongation [106].

### 3.3. DNA Repair at Telomeres

#### 3.3.1. Telomere Uncapping and DSB Repair

Telomeres need protection from DNA degradation and from being recognized as DNA ends by the DSB repair machinery, whose action would lead to chromosomal end-to-end fusions and genetic instability [107,108,109]. Chromosome ends with dysfunctional telomeres are recognized as sites of DNA damage, leading to the activation of the DNA damage response (DDR) which can typically be observed as telomere-induced DNA damage foci (TIF)—for instance, colocalized γH2AX and TRF2 foci [110,111]. Through its capping of telomere ends and interaction with DDR factors, the shelterin complex exerts a crucial role in protecting telomeres, controlling telomeric DNA end resection and repressing the DNA damage response and DSB repair pathways that threaten telomeres [68,110]. Telomere fusions elicited by their uncapping can be mediated by classical NHEJ (e.g., upon loss of TRF2) and alternative end-joining (e.g., upon loss of POT1) [112,113]. In addition to shelterin factors, TERRA lncRNAs have also been shown to protect telomeres (refs [72,75,77] but see also Silva et al. [106]). 

Although HR at telomeric ends is suppressed in normal human cells and telomerase-positive cancer cells [68], Mao et al. showed that the repair of DSBs introduced within the telomeres of proliferating cells by the CRISPR/cas9 technique involved DSB end-resection and occurred through RAD51-dependent HR, predominantly between sister chromatids and, more rarely, between non-sister chromatids [114] (Figure 1).

#### 3.3.2. Base Excision Repair

Given the reactivity of guanines toward oxidative stress [115,116,117], their abundance in telomeric repeats, and their propensity to generate G-quadruplex (G4) structures that impact telomerase and DNA replication [108,118,119,120], telomere protection is especially required against oxidative guanine damage. Guanine oxidation lesions can be induced by reactive oxygen species generated as by-products of cellular metabolism or upon exposure to DNA damaging agents, and their repair in mammals is primarily achieved by the BER pathway [121,122,123]. Richer and von Zglinicki described a continuous correlation between oxidative stress and telomere shortening in fibroblasts [124]. Likewise, telomere erosion induced by high oxidative stress was observed in mouse hematopoietic cells and primary MEFs carrying a homozygous deletion of the DNA glycosylase gene Ogg1 [125]. 

Several groups have developed chemoptogenetic approaches to induce localized oxidative damage (e.g., 8-oxoG) on telomere DNA and monitor the real-time damage response at the single telomere level in ALT-positive and telomerase-positive cells. These studies identified NTH1 [126] and OGG1 [127] as crucial DNA glycosylases in the repair of telomeric oxidative damage while showing that such damage induced cell senescence and cell death, and was a potent inducer of telomere attrition and genomic instability. Of note, Sun et al. provided evidence that ALT cells are more vulnerable to oxidative telomere damage than telomerase-positive cells due to a dependency on recombination for maintaining telomere length [126]. Importantly, unrepaired telomeric 8-oxoG lesions disrupted DNA replication, triggering the repair of collapsed replication forks by MiDAS or RAD51-dependent HR [127]. Also involved in the removal of guanine oxidation lesions at telomeres is NEIL3, a member of the Nei-like (NEIL) DNA glycosylase family [128,129,130] that is recruited to telomeres by TRF1 during S phase [128]. Notably, NEIL3 association with telomeres increases upon oxidative stress [128] while NEIL3 gene expression itself undergoes upregulation upon oxidative base damage [131]. Finally, recent studies reviewed by Kumar et al. have revealed an interplay between BER and NER factors in the repair of oxidative DNA lesions, with specific NER proteins including UV-DDB1—involved in DNA damage recognition during global genome NER—operating to stimulate certain DNA glycosylases involved in the removal of oxidative base damage [132]. Interestingly, the authors showed that UV-DDB1 was recruited to telomeric 8-oxoG lesions prior to OGG1, indicating a direct role for UV-DDB as an early damage sensor in telomeric chromatin, acting upstream of BER [132].

## 4. The *ATRX*/DAXX Histone H3.3 Chaperone in Chromatin Dynamics

**Figure 3:**
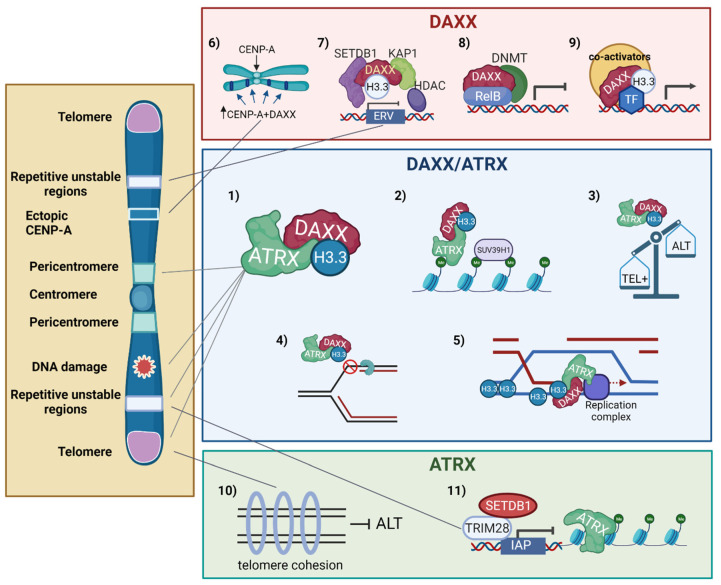
Functions of the *ATRX*/DAXX histone H3.3 chaperone complex and mutually independent functions DAXX and *ATRX* in chromatin dynamics, DNA repair, and genomic stability. Central Panel: The *ATRX*/DAXX complex deposits H3.3 at heterochromatic loci with underlying repetitive DNA elements, including telomeres and (peri)centromeres (1). In cells undergoing global demethylation, *ATRX*/DAXX recruits the SUV39H1 histone methyltransferase to foster the heterochromatinization of specific tandem repetitive elements (2). *ATRX*/ADXX also prevents ALT development (3) and replication stress associated with the formation of secondary structures in repetitive DNA sequences (4). Finally, *ATRX*/DAXX-mediated deposition of H3.3 promotes D-loop stabilization and repair synthesis during DSB repair by HR (5). Other DNA repair functions are detailed in the text. Top panel: *ATRX*-independent functions for DAXX include the ectopic deposition of over-expressed CENP-A (6) and roles as a transcriptional activator or repressor depending on the context (7–9). In association with SETDB1, KAP1, and HDAC1, DAXX is responsible for the silencing of ERVs (7). DAXX recruitment to RelB-regulated genes contributes to their transcriptional silencing mediated by the DNA methyltransferase DNMT1 (8). DAXX may also exert transcriptional repression roles through interaction with HDACs (not illustrated). DAXX can also serve as a transcriptional co-activator in certain situations, such as during heat-induced activation of stress response genes (9). On its part, *ATRX* maintains cohesion between sister telomeres (10) and exerts a KAP1/TRIM28- and SETDB1-mediated repression of IAP retrotransposons by reinforcing the heterochromatin signature at these sites (11). See text for a full description of the DNA repair functions of H3.3/*ATRX*/DAXX. Created with BioRender.

The functions of *ATRX* and DAXX in the chromatin landscape, as well as the functional modules they harbor, and the mutations/alterations observed in cancer have been reviewed recently [15,133,134,135]. The functions assigned to the *ATRX*/DAXX histone H3.3 chaperone complex, as well as the mutually independent functions of DAXX and *ATRX* in chromatin dynamics, DNA repair, and genomic stability are summarized in Figure 3 and detailed below.

In the *ATRX*/DAXX complex, DAXX provides the H3.3-specific chaperone activity. *ATRX* is a chromatin remodeling protein that contains a C-terminal SWI/SNF2-type ATPase/helicase domain as well as several reader modules of epigenetic marks [134], including an atypical PHD finger domain that serves as an H3-binding module whose binding is promoted by lysine 9 trimethylation (H3K9me3) combined with the absence of H3K4 methylation [136,137]. This domain targets DAXX-dependent H3.3 deposition at H3K9me3-enriched heterochromatin. The *ATRX*/DAXX histone chaperone complex deposits H3.3 at heterochromatic repetitive regions including (peri)centromeres, telomeres, and transposable elements such as endogenous retroviral elements (ERVs). The centromere is a chromosomal locus epigenetically defined by the presence of the histone H3 variant CENP-A, which plays crucial roles in the assembly and function of the kinetochore and the maintenance of sister chromatid cohesion during cell division [138,139]. Centromeres are flanked by large heterochromatic regions—the pericentromeres—that play an important role in fostering sister chromatid cohesion during mitosis [140,141]. Like telomeres, the megabase-long chromosomal regions harboring (peri)centromeres are comprised of repetitive DNA sequences [139]. In addition, like telomeres, (peri)centromeric regions are transcribed into lncRNAs that participate in the establishment/maintenance of heterochromatin in these regions [138,142]. Importantly, (peri)centromeric repeat sequences can generate secondary structures upon unwinding of the DNA helix—e.g., during transcription—which hampers the progression of the DNA replication fork and threatens genome stability [139,143,144]. Such structures include stem loops, as well as DNA:RNA hybrids (R-loops). At centromeres, the resolution of DNA secondary structures has been shown to involve the DNA2 nuclease/helicase [145] while BRCA1 prevents R-loop-associated instability [146]. Of note, Tsouroula et al. have revealed the complexity of repairing DSBs generated at pericentric or centromeric heterochromatin [147]. Additional sources of centromere breaks include segregation errors during mitosis, as well as DNA decatenation problems. (Peri)centromeres are thus a source of chromosomal instability, with profound implications for human diseases and cancer [139,148]. 

He et al. have shown that the *ATRX*/DAXX complex protects repetitive elements during global DNA hypomethylation by promoting heterochromatinization of these elements through SUV29H histone methyltransferase recruitment and H3K9 trimethylation [149]. The importance of *ATRX*/DAXX-mediated H3.3 deposition at telomeres and (peri)centromeres has been demonstrated by Jang et al., who showed that H3.3 depletion caused dysfunction of the heterochromatin structures at telomeres and (peri)centromeric regions, resulting in a more open structure in these regions, and mitotic defects [150]. At centromeres, nucleosomes containing H3.3 have been proposed to serve as placeholders during S phase to ensure the subsequent assembly of CENP-A in G1 [151]. *ATRX*/DAXX-mediated H3.3 deposition within (peri)centric heterochromatin has been shown to modulate transcription of the (peri)centric DNA repeats in mice [12]. Finally, the evidence also indicates that H3.3 deposition by the *ATRX*/DAXX complex also takes place in the gene body of specific genes, e.g., to facilitate transcription through regions containing guanine-rich sequences, predicted to form G4 quadruplex structures [152]. 

In addition to forming an H3.3 chaperone complex, *ATRX* and DAXX have each evolved mutually independent functions in gene expression regulation, chromatin dynamics and DNA repair [15,133,134,135]. DAXX mediates the ectopic deposition of CENP-A in cancers where this centromeric histone variant is overexpressed [138,153]. As ectopic CENP-A can recruit components of the kinetochore, this process is potentially associated with chromosome segregation aberrations and chromosome instability. DAXX also functions as a gene-specific transcriptional co-repressor [133], recruiting DNA methyltransferase 1 (DNMT1) [154] or histone deacetylases [155] to foster epigenetic silencing. DAXX can also serve as a transcriptional co-activator in certain situations, such as during the heat-induced activation of stress response genes [156]. DAXX also exerts an *ATRX*-independent role in the silencing of endogenous retroviruses (ERVs) [157,158], while *ATRX* has evolved a similar DAXX-independent function in the repression of intracisternal A particle (IAP) retrotransposons [159]. Finally, *ATRX* was shown to possess both DAXX-dependent and DAXX-independent roles in controlling DSB repair pathway choice at telomeres (ref [40] and see below).

## 5. Impact of Mutations in H3.3/*ATRX*/DAXX on DNA Repair and Telomere Dynamics

### 5.1. H3.3 and Its Chaperones in DNA Repair

Shortly following UV-induced DNA damage in human cells, H3.3 is rapidly deposited by HIRA at sites of UV lesions where it primes damaged chromatin for reactivation of transcription following NER-mediated repair, suggesting that HIRA-dependent histone deposition serves as a chromatin bookmarking system to facilitate transcription recovery after genotoxic stress [160]. Frey et al. found that chicken DT40 cells completely lacking H3.3 were hypersensitive to UV light, a defect suggested to result from less-effective nucleotide excision repair [161]. Notably, H3.3 knockout cells were not hypersensitive to X-rays but exhibited mild hypersensitivity to the intra- and inter-strand crosslinking agent cisplatin and the alkylating agent methyl methanesulfonate (MMS), suggesting a broad requirement for H3.3 in facilitating DNA repair. Interestingly, the cancer-associated mutations K27M, G34R, and G34V all conferred a UV-sensitive phenotype similar to the H3.3 knockout, suggesting a role for these residues in facilitating NER and the possibility that, in addition to affecting transcriptional regulation, cancer-associated H3.3 mutations could impact DNA repair. Notably, the authors identified a requirement for H3.3 in maintaining replication fork progression on UV-damaged DNA, a process that was independent of NER [161]. Finally, Huh et al. [162] have shown that loss of *ATRX*/DAXX in HeLa cells increased their sensitivity to replication stress induced by hydroxyurea (HU), which depletes the dNTP pool and causes fork stalling (upon short exposure) or collapse (upon long exposure) [163]. They also observed that protection of stalled replication forks was inefficient in these cells, leading to genomic instability. The authors proposed a model whereby deposition of H3.3 helps prevent secondary DNA structures that hamper replication, while *ATRX* also prevents excessive resection of stalled resection forks, thus facilitating their restart by RAD51-mediated mechanisms [162]. 

In response to DSBs, Luijsterburg et al. showed that PARP1-mediated recruitment of the chromatin remodeler CHD2 triggers the rapid deposition of H3.3 at sites of DNA damage to create a chromatin microenvironment promoting repair by NHEJ [164]. Moreover, Li and Tyler showed that chromatin reassembly following NHEJ-mediated DSB repair was dependent both on HIRA (specific to H3.3) and CAF-1 (specific to H3.1/H3.2), suggesting that epigenetic information is restored by the concerted action of replication-independent and replication-dependent chromatin assembly pathways [165]. 

*ATRX*-deficient GBM tumor cells were found to be hypersensitive to DNA damaging agents inducing DSBs [166]. Juhasz et al. [167] examined the repair of laser-induced DSBs in G2-phase cells and H3.3 dynamics in vivo. They observed that newly-synthesized H3.3 was deposited at sites of DNA damage in a HIRA-dependent manner at early time points following DSB induction, which corresponded to rapid repair events mediated by NHEJ [168], in line with the findings of Luijsterburg et al. [164]. In addition, H3.3 was also deposited at late time points corresponding to a slower, HR-mediated repair, and such deposition was dependent upon *ATRX*/DAXX. Furthermore, *ATRX*/DAXX-mediated deposition of H3.3 at DSB sites promoted DNA repair synthesis and sister chromatid exchange during HR [167]. Specifically, H3.3 deposition occurred downstream of the RAD51 removal step that follows strand invasion and D-loop formation, promoting classical DSBR (Figure 1). Interestingly, cells lacking *ATRX*, DAXX, or H3.3 showed a defect in repairing IR-induced DSBs by HR. In addition, loss of *ATRX* abolished extended DNA repair synthesis and sister chromatid exchanges at the induced DSBs, instead favoring SDSA (Figure 1 and Figure 3) [167]. The authors hypothesized that *ATRX*/DAXX-mediated deposition of H3.3 at sites of repair synthesis may be required to overcome topological constraints at the moving D-loop [167,169]. Finally, Chu et al. have analyzed the non-telomeric targets of the TERRA lncRNAs, revealing that TERRA and *ATRX* share hundreds of target genes where their effect on gene expression is functionally antagonistic [72]. As TERRAs have been shown to undergo post-transcriptional regulation and stabilization in response to DNA damage including DSB-forming agents [170], it will be illuminating to learn if and how the TERRA-*ATRX* antagonism affects gene expression in response to DNA damage.

How the H3.3 (K27M) and H3.3 (G34R/V) mutations can alter DNA repair capacities in pediatric GBM is starting to emerge, in part thanks to studies in non-mammalian eukaryotic models, revealing indirect as well as direct effects. Lewis et al. found that the H3.3 (K27M) mutation reduced the levels of H3K27me2/H3K27me3 marks deposited by the enhancer of teste homologue 2 (EZH2)—the catalytic subunit of polycomb repressive complex 2 (PRC2)—through interaction with EZH2 and inhibition of PRC2 activity [171]. De Vries et al. observed that prolonged depletion of EZH2 in mouse-derived glioblastoma stem-like cells (GSCs) led to global transcriptional reformatting and increased tumor progression [172]. Importantly, gene ontology (GO) analysis identified “DNA repair” as a major enriched term in EZH2-depleted GBMs, with key HR factors including RAD51 upregulated. Thus, increased resistance to TMZ driven by RAD51-mediated DSB repair was observed following EZH2 inhibition in GBM cells. The authors suggested that concomitant inhibition of EZH2 and HR (e.g., RAD51) might potentiate TMZ toxicity in GBM [172]. As EZH2 is overexpressed not only in adult GBM but also in pHGGs [173] where RAD51 is also found overexpressed [174], such a strategy may also be proposed for the H3.3/H3.1 (K27M) subgroups of pHGGs. Delaney et al. revealed that the introduction of the H3.3 (K27M) mutation into *C. elegans* affected PRC2 activity and elicited alterations in H3K27me3 distribution and gene expression [175]. In addition, the H3.3 (K27M) mutation resulted in the dysregulation of specific serine/threonine kinases such as KGB-1, a homolog of mammalian Jun amino-terminal kinase (JNK)—itself a known target for inhibiting proliferation in GBM cells [176,177,178], leading to ectopic activation of DNA replication, DNA damage accumulation, and endomitotic oocytes in proximal germ cells. Importantly, the authors found that pharmacological inhibition of JNK suppressed the proliferation of pHGG cells derived from K27M tumors more strongly than cells derived from non-K27M pHGG cells [175]. 

The H3.3 (G34/R/V) mutations impair SETD2 catalytic activity and block H3K36 methylation in vitro and in vivo [8,171,179]. SETD2-dependent H3K36me3 is crucial for DNA repair, including DSB repair by HR [180,181,182], and also modulates mismatch repair (MMR) through its interaction with the MMR recognition protein MutSα [183]. Fang et al. found that the H3.3 (G34/R/V) mutations led to MMR deficiency and a mutator phenotype by preventing H3K36 trimethylation involved in the recruitment of MutSα to chromatin and/or directly reducing the interaction between H3K36me3 and MutSα [179]. Notably, mining of the whole-genome sequencing dataset of pediatric gliomas in the International Cancer Genome Consortium database revealed that the H3.3 (G34R) mutation was associated with a significant increase in somatic mutation compared to a non-G34 mutation group, including the H3.3 (K27M) mutation [179]. 

Insights into the repair functions of H3.3 (G34R) were also provided by the analysis of fission yeast cells, which normally express three genes encoding a single histone H3 that has features of both human H3.1 and H3.3 histones. Specifically, Yadav et al. engineered yeast cells to express H3 (G34R) as their only H3 histone [184]. Their results show that H3 (G34R) expression increased genetic instability, including enhanced chromosome loss and defects in chromosome segregation. In addition, H3 (G34R) expression led to increased replication stress and slowed the replication fork restart, although DNA replication checkpoints were functional. Of note, in line with the data of Frey et al. suggesting that cancer-associated H3.3 mutations could impact DNA repair [161], H3 (G34/R) cells displayed hypersensitivity to agents generating replication stress and replication fork collapse (including HU, MMS, and CPT). In addition, H3 (G34R) cells were defective in HR but not NHEJ [184]. At the mechanistic level, it was suggested that reduced levels of H3K36me3 and H3K36Ac observed in H3 (G34R) cells may affect nucleosome removal and DNA end resections at broken replication forks, leading to delayed HR repair and genomic instability [184]. 

In follow-up work, Lowe et al. showed that the H3 (G34V) mutation, although reducing the levels of H3K36me3 in a similar way as H3 (G34/R), elicited distinct other phenotypes [185]. Thus, the H3 (G34V) mutant showed no defect in H3K36 acetylation, no chromosome loss, no replicative stress, and no defects in HR, but enhanced sensitivity to IR. It also repressed subtelomeric gene transcription. Notably, reintroduction of wild-type H3 into H3 (G34R) or H3 (G34V) revealed that only some defects, including the HU sensitivity and HR defects in H3 (G34R) mutants, and silencing of subtelomeric domains in H3 (G34V) mutants, were dominant. In view of the phenotypes manifested in the fission yeast, including the distinct DNA damage phenotypes of the different H3G34 mutations, the authors pointed out that “It will be interesting to determine the extent to which the phenotypes uncovered in this study are shared in mammalian cancer cells expressing these mutants [185]”.

Finally, it is noteworthy that the H3.3 and H3.1 mutant histones can be found associated with genetic alterations/mutations expected to perturb DNA repair (reviewed by Pedersen et al. [22] and Metselaar et al. [21]). For instance, Mackay et al. [3] reported that 60% of the 1000 pHGGs characterized in their integrative genomic analysis contained genetic alterations in DNA repair genes, including HR genes and Fanconi anemia genes involved in the protection/restart of replication forks and DNA crosslink repair [186]. 

### 5.2. H3.3 and Its Chaperones in Telomere Dynamics and Telomeric DNA Repair

H3.3. mutations and loss of *ATRX* and DAXX are associated with ALT activation. Recently, Minasi et al. have investigated ALT in a cohort of 52 IDH-wildtype, 1p/19q-wildtype pHGGs exhibiting mutations in H3F3A, *TERT* promoter (*TERTp*), and/or *ATRX* [91]. Mutant H3.3 was present in 40.3% of the cases with an almost equivalent distribution of K27M (19.2%) and G34R (21.1%) mutations. Corroborating previous studies [4], 100% of the H3.3 (G34R)-mutated pHGGs displayed the ALT phenotype while a much lower incidence rate was observed in H3.3 (K27M) pHGGs (40%). Similarly, *ATRX* nuclear loss always triggered ALT and was sometimes associated with the G34R mutation but never with the K27M mutation. These data suggest that, unlike H3.3 (K27M), H3.3 (G34R) always triggers ALT, irrespective of *ATRX* status. 

Ectopic expression of *ATRX* or DAXX suppressed ALT features (e.g., APBs, C-circles) in ALT-positive, *ATRX*- or DAXX-deficient cell lines [187,188,189]. Several scenarios have been proposed to explain the participation of *ATRX* and/or DAXX in ALT suppression. The evidence suggests that HR-mediated repair of stalled replication forks is a key determinant of the ALT pathway [190,191,192,193]. Because of their impact on replication and repair [108,120,127,194], telomeric G4 structures have been proposed to play a crucial role in ALT suppression by *ATRX*. Thus, Clynes et al. found that ectopic re-expression of *ATRX* in the *ATRX*-null, ALT cell line U2OS reversed the ALT phenotype including the levels of C-circles and APBs, increased the levels of telomeric H3.3, and reduced replication fork stalling [188]. In line with studies by Law et al. showing that *ATRX* can recognize/modulate telomeric G4 structures in vitro [195], the authors also found that treatment with a G4-stabilizing ligand known to induce telomere dysfunction impeded the ability of *ATRX* to suppress ALT. Finally, they presented data suggesting the involvement of *ATRX* in inhibiting telomeric recombination. [188]. The authors, therefore, proposed that while *ATRX*/DAXX-mediated H3.3 deposition at telomeres helps maintain a telomeric DNA free of G4 structures, thus facilitating DNA replication and maintaining genomic stability, loss of *ATRX*/DAXX causes replication fork collapse at these structures, triggering HR and ALT at telomeres [188]. This model is supported by recent work from Teng et al. who confirmed the interaction of *ATRX* with G4 structures and showed that H3.3 deposition by *ATRX*/DAXX helps maintain a heterochromatin state that protects cells from G4-mediated stress [196]. 

DNA:RNA hybrids (R-loops) resulting from TERRA transcription are more frequent in ALT-positive cells than in telomerase-positive cells [197]. Notably, Yang et al. recently established an interdependent relationship between ALT-associated G4s and R-loops, confirming that these two structures can be spatially linked into unique, highly stable structures called G-loops, at the telomeres [197]. Interestingly, work by Nguyen et al. suggested that *ATRX* recruitment to G-rich telomeric repeats may occur through the formation of R-loops exposing G4 structures [198]. The authors proposed that *ATRX*/DAXX-mediated H3.3 deposition promotes R-loop resolution and the dissolution of G4 structures during DNA replication. As telomeric R-loops are one of the substrates for HR in ALT [199], they also suggested that *ATRX* prevents the triggering of ALT by suppressing recombinogenic R-loops [198]. 

Work by Li et al. [200] also implicated ALT activation as an adaptive response to telomere replication dysfunction induced by *ATRX*/DAXX but suggested that *ATRX* and DAXX do not act as direct ALT suppressors. Rather, the authors showed that by causing chromatin decompaction, loss of *ATRX*/DAXX gradually induced telomeric DNA replication stress, activating DDR and homology-directed DNA repair including ALT. Notably, ChIP analyses revealed a decrease in the levels of telomeric H3.3 in the *ATRX*-depleted cells. In addition, telomere dysfunction phenotypes similar to those elicited by *ATRX* depletion were obtained following the depletion of DAXX; these could be rescued by wild-type DAXX but not by mutants impaired in their ability to interact with *ATRX* or H3.3 [201], linking the association of the telomere defects with impaired H3.3 deposition by the histone chaperone complex *ATRX*/DAXX [200]. The authors pointed out that the late emergence of ALT features observed upon the loss of *ATRX*/DAXX in their experiments was consistent with observations in pancreatic neuroendocrine tumor biopsies of different stages by Marinoni et al. [166], indicating that the loss of *ATRX* or DAXX occurred well before ALT development. Finally, the authors also established that endogenous telomerase activity cannot overcome the telomere defects induced by *ATRX* deficiency and that *ATRX* mutant cells are forced to adopt ALT during immortalization [200].

Recently, Lovejoy et al. [40] have presented data supporting a model whereby *ATRX*, independently of DAXX and by a yet-unknown mechanism, maintains sister telomere cohesion at telomeric DSBs, thus preventing ALT by averting unequal (out-of-register) sister telomere recombination and the use of non-allelic telomeres for DSB repair. Instead, the loss of cohesion at telomeric DSBs triggered by *ATRX* disruption would favor such interactions, which are necessary to extend telomeres and are in line with recombination events seen in ALT cells. Notably, the full spectrum of telomeric DSB repair defects associated with *ATRX* disruption could be recapitulated by the combined loss of DAXX and telomere cohesion—induced through disruption of stromal antigen 1 (SA1), a member of the canonical cohesin complex—but not by the sole disruption of SA1. The authors, therefore, suggested that ALT promotion also involves disruption of a DAXX-dependent function of *ATRX* [40]. 

In summary, H3.3 and its chaperones facilitate DNA repair (including NER, MMR, NHEJ, and HR), as well as post-repair chromatin reconstitution. In addition, they are important for the prevention of replication stress. The evidence indicates that the impact of mutations affecting H3.3/*ATRX*/DAXX on DNA repair is both direct and indirect. Notably, *ATRX*/DAXX also facilitates chromatin reconstitution during the DNA repair synthesis step of HR, thus dictating which subpathway is used to repair DSBs. Likewise, loss of H3.3/*ATRX*/DAXX integrity is associated with the development of ALT mechanisms of telomere elongation that mobilize crucial DNA repair/recombination factors.

## 6. Strategies Targeting Chromatin Dynamics and DNA Repair in pHGGs

How the genetic and epigenetic alterations defining each pHGG subgroup provide opportunities for improving patient survival constitutes a formidable challenge that is compounded by the scarcity of pHGG models available for in vitro and in vivo studies. 

Several clinical trials have been initiated to test therapeutic strategies against the various pHGG subgroups [202,203]. In many instances, such strategies target molecular alterations that cosegregate with specific histone mutations. For tumors defined by the H3.3 (K27M) mutation, PDGFR inhibitors have been proposed, since PDGFRA (platelet-derived growth factor A) amplification frequently cosegregates with the H3.3 (K27M) mutation [3,204]. On the other hand, inhibitors of the tropomyosin receptor kinase (TRK) family of tyrosine receptor kinases [205] are being tested against tumors defined by the H3.3 (G34R/V) mutations where recurrent fusions involving the TRK genes are observed [7,202]. 

**Figure 4:**
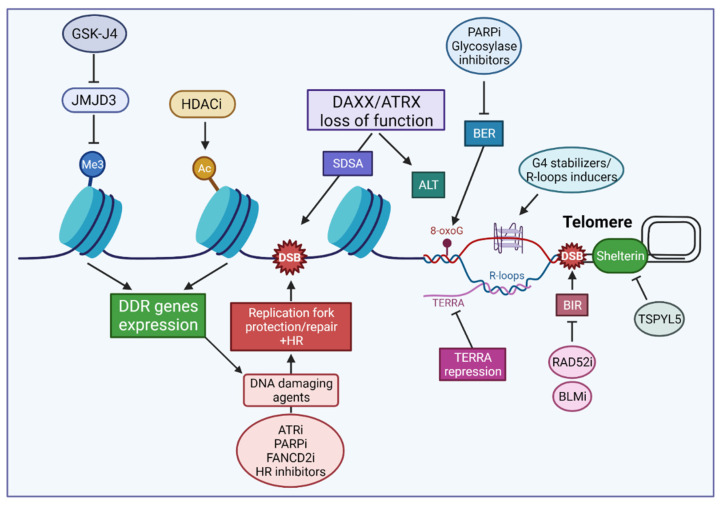
Therapeutic strategies targeting DNA repair and telomere dynamics in pHGGs. Illustrated are current or proposed strategies affecting the expression of DNA repair genes through the targeting of epigenetic modifiers operating at marks impacted by the oncogenic histone H3.1/H3.3 mutations, or exploiting the replication stress inherent to pHGG cells, alone or in combination with DNA damaging agents. Other potential targets include components of the SDSA pathway, which is the favored HR DSB repair pathway upon loss of *ATRX*/DAXX. Also illustrated are strategies targeting telomeric DNA repair and telomere maintenance mechanisms. See text for details. Created with BioRender.

Recently, strategies have emerged that use inhibitors targeting the epigenetic changes associated with mutations affecting the histone H3 variants, either alone or in combinatorial treatment. Leszczynska et al. have assessed the effectiveness of epigenetic drugs in the context of DIPGs carrying the H3.1 (K27M) or H3.3 (K27M) mutations [206]. As these mutations decrease the global levels of H3K27me3 and increase H3K27ac, efforts have been devoted to targeting epigenetic modifiers of these marks. Here, we will focus on those approaches that impacted DNA repair (Figure 4). Notably, one study aiming at restoring the K27me3 repressive mark demonstrated that pharmacological inhibition of the K27 demethylase JMJD3 by GSK-J4 displayed potent antitumor activity in vitro against H3.3 (K27M) cells and extended the survival of mice bearing H3.3 (K27M) tumors [207]. Moreover, GSK-J4 was found to inhibit the expression of several DNA repair genes in H3.3 (K27M) mutant DIPG cells, and it sensitized these cells to IR both in vitro and in orthotopic human DIPG xenografts [208]. However, Leszczynska et al. noted that its rapid conversion to GSK-1J puts a constraint on the use of GSK-4J in clinical trials [206]. Several strategies targeting HDACs have also been considered in DIPGs, notably in combination with inhibition of the AXL kinase, one of the initiators of the epithelial to mesenchymal transition signature observed in DIPG tumors [206]. Thus, the HDAC inhibitor panobinostat was found to radiosensitize DIPG cells, and this effect was increased in the presence of the AXL inhibitor BGB324. Notably, the combination of panobinostat and BGB324 led to a decrease in DNA repair gene expression, including FANCD2 and RAD51 [209]. Several studies have reported that DNA repair factors represent possible HDAC targets and that HDAC can sensitize cancer cells to IR and other anticancer agents [210,211,212]. It is notable that, while GSK-J4 was recently shown to exert a protective effect in Parkinson’s disease models in vivo, confirming its ability to cross the blood brain barrier (BBB) [213], the testing of panobinostat in mice with DIPG xenografts required convection-enhanced delivery past the BBB [209]. To date, the ability to cross the BBB and the development of adequate techniques to deliver therapeutics directly to the brain remain major hurdles in testing the therapeutic efficacy of drugs against tumors of the central nervous system [214,215,216].

Genomic instability induced by defects in DNA repair/chromatin dynamics is a major driver of tumorigenicity [217]. A growing body of evidence indicates that many cancers have acquired DNA repair defects that render them addicted to rescue repair pathways in order to cope with oncogene activation and the burden of DNA damage associated with high proliferation, metabolic and signaling aberrations, or genotoxic treatment [218,219,220]. Targeting DNA repair pathway addictions, through inhibition of components of the DDR, including modulation of cell cycle and mitotic progression, and genetic stability, has emerged as an important therapeutic approach against many cancers [221,222,223,224]. At the same time, our catalogue of small molecule inhibitors targeting DNA repair is expanding rapidly [225,226,227,228] while novel targets are being discovered for the sensitization of glioma cells to radio- and chemotherapy [21,22]. These include RAD52 whose depletion led to TMZ hypersensitivity in GBM cells [64]. Carruthers et al. found that adult GBM stem-like cells display high levels of DNA replication stress driving constitutive DDR activation and radiation resistance [229]. The authors showed that targeting replication stress by combined inhibition of ATR—the apical DNA replication stress kinase [230]—and PARP1 conferred GSC-specific cytotoxicity and abrogated GSC radiation resistance completely in vitro [229]. As underlined in this review, there is a strong connection between mutations in H3.3/*ATRX*/DAXX and DNA replication stress. This is illustrated, e.g., by the hypersensitivity to hydroxyurea and genomic instability associated with loss of *ATRX*/DAXX or the oncogenic mutations affecting H3.3. Notably, indicative of a protective response induced by stalled/collapsed replication forks, PARP1 was found to be hyperactivated in *ATRX* KD mouse cells, while its inhibition elicited DSBs and suppressed growth in these cells [162]. Additional evidence for the importance of a DNA replication stress response in pHGGs was also provided by Metselaar et al. who observed the specific overexpression of the Fanconi core component FANCD2—a critical actor in the protection/restart of replication forks and DNA crosslink repair—in a cohort of pHGGs [231]. Revealing the dependency of pHGG cells on FANCD2, the authors found that induction of FANCD2 proteasomal degradation by the BBB-penetrable, plant derivative celastrol, or its depletion via RNAi, sensitized pHGG cells to the DNA crosslinking agents carboplatin [231]. At the mechanistic level, celastrol was found to induce genomic instability by causing replication fork arrest, probably caused by a FANCD2 depletion-induced loss of RAD51 expression. While the in vivo investigation of H3.1/H3.3-mutated DIPG pre-clinical models remains to be achieved, the authors noted that combining celastrol and carboplatin prolonged survival in a pHGG xenograft model (cortical GBM, wild-type H3.3/H3.1, mutant *BRAF*) [231]. 

In line with the highly-proliferative phenotype of pHGG cells and the importance of RAD51 in DNA replication protection and the recovery of stalled replication forks [232], Entz-Werlé et al. [174] have reported the overexpression of RAD51 protein in pHGGs. When analyzed at the protein expression level, the authors also reported that PARP1 and XRCC1, as well as KI67, were significantly overexpressed in a subgroup of highly radio-resistant pHGGs displaying very early relapse; this high expression correlated with a worse OS. Interestingly, high PARP1 expression level also stood out as a biomarker associated with histone mutated tumors [174]. That high PARP1 expression is associated with poor prognosis was also observed by van Vuurden et al., who also showed that PARP1 inhibition sensitized pHGG to IR [233]. Current studies testing PARP1 inhibition, in combination with TMZ or IR, in pHGG have been reviewed [234]. In this regard, it is noteworthy that the H3.3 (G34R/V) mutations led to MMR deficiency [179]. Indeed, proficient MMR is required for unrepaired O6-meG lesions induced by TMZ to generate cytotoxic seDSBs, and the emergence of MMR deficiencies is a documented scenario associated with TMZ resistance in gliomas [235,236,237], suggesting that defective MMR may contribute to TMZ resistance in H3.3 (G34/R/V) pHGGs. As PARP1 inhibition has been shown to restore TMZ sensitivity in MMR-deficient GBMs [238], we propose that such inhibition may help sensitize H3.3 (G34/R/V) pHGGs to alkylating agents. Finally, it is notable that the integrity of *ATRX*/DAXX dictated which HR pathway was used to repair IR-induced DSBs, with SDSA being favored upon the loss of *ATRX*/DAXX [167]. Recently, Elbakry et al. revealed that the SDSA subpathway depended on RECQ5 [239]. As our knowledge of the DNA repair factors that promote or modulate SDSA is improving [240], future strategies may target SDSA components to promote radiosensitivity in *ATRX*/DAXX-deficient cells. 

Fan et al. have previously reviewed approaches targeting telomerase or the ALT pathway to trigger cell death in GBM, including mutant H3.3/*ATRX*/DAXX tumors [241]. Several G4-interacting molecules showing specificity for telomeric G4s have been developed (reviewed in ref [242]). Through their ability to induce replication stress, DNA damage, and hamper DNA repair at telomeres, G4 ligands may provide potent anti-cancer drugs in pHGGs. In this regard, Yang et al. noted that, in ATL cells, such G4 ligands, combined with R-loop inducers, may lead to G-loop hyperstabilization, thus contributing to a hyper-ALT phenotype characterized by the accumulation of toxic recombination intermediates [197]. Several of the BER mouse knockouts lead to embryonic lethality, suggesting that BER inhibitors may be toxic to noncancerous cells. However, this is not the case for DNA glycosylases, with the exception of thymine-DNA glycosylase (TDG) [243]. Although drugs that target DNA glycosylases are still in development [244], targeting the multiple DNA glycosylases involved in the repair of oxidative DNA damage may represent an attractive approach, in particular to challenge telomere integrity. Recently, Episkopou et al. identified the USP7 deubiquitinase inhibitor TSPYL5 (testis-specific Y-encoded-like protein 5) as the first molecular, ATL-specific-target. The authors demonstrated that ALT telomeres need to be protected from the degradation of the shelterin component POT1 in APBs, and identified TSPYL5 as a PML resident that colocalizes with ALT telomeres and protects POT1 from proteosomal degradation exclusively in ALT-positive cells [245]. Although it remains to be seen whether/how the mechanism identified by the authors is impacted by the various mutations affecting the H3.3/*ATRX*/DAXX complex in the pHGG subgroups, these results provide strong support for the development of therapeutic strategies targeting ATL-positive cells through debilitating their TMM. In this regard, the factors that mediate BIR represent attractive therapeutic targets, including the RAD52 recombinase and the RecQ DNA helicase BLM that participates in both RAD52-dependent and independent BIR pathways [105]. As RAD52 inactivation induces synthetic lethality in cells harboring deficiencies in key HR factors [246,247,248,249] and several RAD52 inhibitors have been developed [227,250], we propose that the targeting of RAD52 is a particularly attractive target in ALT pHGGs. Finally, given its role in destabilizing telomere integrity and BIR initiation in ALT cells, Silva et al. have proposed that TERRA transcription, through its impact on telomere instability, might become a useful target for therapy [106]. Figure 4 summarizes potential therapeutic strategies targeting DNA repair and telomere dynamics in pHGGs. In summary, the evidence indicates that mutations in H3.3/*ATRX*/DAXX confer an important level of DNA replication stress in pHGG cells, as well as a dependency on DNA repair and recombination pathways that afford protection against replication stress and/or help maintain telomere homeostasis. Although targeting these pathways might represent a promising strategy and while the mechanistic insights summarized in this review have helped identify relevant targets for such strategies, more progress is clearly needed to enable personalized treatment strategies for pHGGs.

Profiling DNA repair and cell cycle gene expression was recently proposed by Gobin et al. as a strategy to classify aGBMs and uncover potential vulnerabilities to therapeutic agents [251]. The authors described a DNA repair and cell cycle gene expression signature that resulted in the classification of aGBM specimens into two major groups (G1 and G3) displaying inverse expression profiles, and a third less-defined group. Of note, G3 tumors were highly proliferative compared to G1 tumors, and the authors presented experimental evidence that the signature exposed group-specific vulnerabilities to DNA repair inhibitors and DNA damaging agents [251]. While Entz-Werlé et al. found that the signature identified in aGBMs could capture features related to cell cycle and DNA repair in pHGG tumors [174], the observed segregations did not correlate with underlying driver mutations affecting histones, as shown in previous studies [2,179,185,252,253,254]. Based on the comparison of transcriptomic datasets from pHGGs and low-grade gliomas, the authors then identified a DNA repair and cell cycle gene expression signature of 28 genes that distinguished pHGGs from pediatric, low-grade gliomas. Notably, this signature was able to cluster the sus-tentorial and thalamic pHGG groups effectively, linking histone mutated tumors to a more proliferative group [174]. Whether this signature exposes therapeutic vulnerabilities to DNA damaging agents and inhibitors of the DNA damage response that can be exploited in precision medicine for pHGG will require more comprehensive genomic and transcriptomic analysis. Likewise, future strategies to identify the specific vulnerabilities—including the DNA repair pathway dependencies—that characterize the various molecular pHGG subgroups are likely to include large-scale genetic screening (e.g., RNAi screens, CRISPR-cas9 screens, and synthetic lethality screens) as well as high-throughput drug screening (e.g., drug repositioning screens). These strategies rely heavily on the availability of models that recapitulate the complexity and heterogeneity of the various pHGG molecular subgroups, which are currently a limited resource. In this regard, recent work by He et al. reporting the establishment and molecular characterization of a number of patient-derived orthotopic models and matched cell lines from diverse pHGG subgroups, and the results of a drug screen, may pave the way toward the development of personalized medicine for pHGG [255].

## 7. Conclusions

In subsets of pHGGs, specific mutations affecting key regulatory residues in the N-terminal tail of histone H3.3 elicit distinct epigenetic reprogramming and oncogenesis scenarios. As summarized in this review, such mutations also affect the DNA repair pathways that remove lesions induced by current radio- and chemotherapies against pHGGs, both indirectly and directly, while affecting the structure and dynamics of heterochromatin regions that are paramount to the integrity of our chromosomes. Such mutations elicit replication stress and are instrumental in promoting telomere maintenance through ALT mechanisms, thus mobilizing key DNA repair and recombination factors. We are only beginning to unravel H3.3/*ATRX*/DAXX mutations and, through them, how specific addictions to DNA repair pathways or chromatin/telomere factors can be exploited to provide novel therapeutic strategies targeting pHGGs. However, current progress in the generation of pHGG subgroup-representative cellular models, combined with the development of small molecule inhibitors of DNA repair and epigenetic factors and advances in drug delivery, should foster the implementation of genetic and high-throughput drug screenings and novel therapeutic strategies to achieve precision medicine for pHGG.
